# Outcome of Solid Organ Transplantation in Patients With Intellectual Disability: A Systematic Literature Review

**DOI:** 10.3389/ti.2024.11872

**Published:** 2024-10-17

**Authors:** Ingeborg de Rover, Lara Orlandini, Sarwa Darwish Murad, Wojciech G. Polak, Jane Hartley, Khalid Sharif, Dimitri Sneiders, Hermien Hartog

**Affiliations:** ^1^ Erasmus Medical Center (MC) Transplant Institute, Department of Surgery, Erasmus Medical Center (MC), Rotterdam, Netherlands; ^2^ Erasmus Medical Center (MC) Transplant Institute, Department of Gastroenterology and Hepatology, Erasmus Medical Center (MC), Rotterdam, Netherlands; ^3^ Liver Unit, Birmingham Children’s Hospital, Birmingham Women’s and Children’s National Health Service (NHS) Foundation Trust, Birmingham, United Kingdom; ^4^ Centre for Liver and Gastrointestinal Research, Institute of Immunology and Immunotherapy, University of Birmingham, Birmingham, United Kingdom

**Keywords:** organ transplantation, intellectual disability, graft survival, rejection, adherence

## Abstract

Access to solid organ transplantation in patients with intellectual disability is associated with health inequities due to concerns about treatment adherence, survival rates, and post-transplant quality of life. This systematic literature review aims to compare outcomes after organ transplantation in patients with intellectual disability compared to patients without intellectual disability. Embase, Medline Ovid, PsycINFO, Web of Science, Cochrane Central Register of Trials, and Google Scholar databases were systematically searched for studies concerning pediatric or adult solid organ transplantation in recipients with a diagnosis of intellectual disability prior to transplantation. Primary outcomes were patient and graft survival rates. Secondary outcomes were acute rejection rate, adherence rates, and quality of life. Nine studies were included, describing kidney (n = 6), heart (n = 4) and liver (n = 1) transplantation. Reported graft survival rates were non-inferior or better compared to patients without intellectual disability, while patient survival was reportedly slightly lower in two studies reporting on kidney transplantation. Although current evidence has a potential selection bias based on including patients with a sufficient support network, intellectual disability alone should not be regarded a relative or absolute contra-indication for solid organ transplantation.

## Introduction

Intellectual disability (ID) as defined by the DSM-5 criteria affects approximately 1% of the general population [1, 2]. ID is associated with increased incidence of concomitant chronic disease and decreased life-expectancy [3]. Additionally, clinicians consider quality of life to be decreased in patients with ID, however when asked, many patients with ID report an acceptable quality of life [4]. Organ transplantation in patients with ID may raise additional concerns, regarding treatment adherence, post-transplant survival benefit, and whether improvement in quality of life after organ transplantation is achievable [5, 6]. Therefore, ID has historically been considered a relative or absolute contraindication for organ transplantation [7, 8].

In the face of organ shortage, transplant benefit and graft utility are important principles guiding access to transplantation and allocation of organs. Along with criteria such as the patients need or urgency and the probability of a successful outcome [9]. However, a report written by the National Council on Disability stated that many transplant centers in North America still have reservations about solid organ transplantation in people with ID: studies from 2006 to 2008 found that 43%–60% of transplant centers considered some degree of ID as an absolute or relative contraindication to transplantation [10]. These assumptions also impacted on a centers’ willingness to evaluate a patient with ID and place them on the waiting list. Approximately one-fifth of transplant centers had formal guidelines for listing candidates with ID and half had informal guidelines [11]. To prevent potential discrimination against people with ID in the allocation of donor organs, decision-making should ideally be based on scientific data, and consensus guidelines would be required.

The present systematic literature review aims to provide an evidence-based analysis of the currently available literature concerning the outcomes of solid organ transplantation in patients with ID, while comparing this to patients without a disability.

## Methods

This systematic literature review was written according to the Preferred Reporting Items for Systematic Reviews and Meta-Analyses (PRISMA) [12]. Additionally, guidelines for synthesis without meta-analysis (SWiM) in systematic reviews were followed [13]. The systematic literature review protocol was PROSPERO registered under registration number CRD42020161607.

### Search Strategy

Comprehensive searches were performed by a biomedical information specialist. Six databases were searched for relevant articles: Embase, Medline Ovid, PsycINFO, Web of Science, Cochrane Central Register of Trials, and Google Scholar (Supplementary Appendix 1). Duplicate entries were removed. Subsequently, unique records were reviewed based on title and abstract by two independent reviewers (IdR, LO). Records selected based on title and abstract were further reviewed for final selection based on the full text article. Disagreement was resolved by consensus with a third reviewer (DS). Finally, manual cross-referencing was performed to identify potentially relevant studies not included in the initial search.

### Study Selection

Original studies were included if they studied pediatric or adult patients with a pre-transplantation diagnosis of ID and compared results to a control group in the setting of solid organ transplantation. Studies were included if they described any of the primary outcomes (graft and patient survival). We excluded case reports and studies discussing ID diagnosed post-transplantation. Studies without an available full text record or written in other languages than English were also excluded.

### Data Extraction and Study Outcomes

Data extraction was performed with a standard extraction table and included study design, type of solid organ transplantation, age, sex, ethnicity, average IQ, definition, assessment, and selection of patients with ID for transplantation, diagnosis regarding ID, and indications for transplantation. The primary outcome of this systematic literature review was defined as the patient and graft survival in solid organ transplantation patients with pre-transplantation diagnosed ID. Episodes of rejection, adherence rates and quality of life were secondary outcomes.

### Quality Assessment

Quality assessment was conducted by two independent reviewers (IdR, LO). The Robins tool, a standard quality assessment tool for non-interventional and observational studies [14], did not differentiate well between the quality of included studies. Therefore, the quality of methodological steps was assessed and summarized for all studies, including source population, case definition, patient selection bias, definition of outcomes and data collection methods. Overall quality of the individual studies was summarized along principles of scope and purpose, design, sampling of the studied cohort, data collection, analysis, validity, generalizability, and credibility.

### Data Synthesis

Outcome data was extracted and grouped per specific organ, and then tabulated or described in the review text. Possible outcomes were described with reference to the accurate definition and classification of the outcome. Survival proportions were given as described by the individual studies or estimated from survival curves as described and validated previously [15].

## Results

### Literature Search Results

**FIGURE 1:**
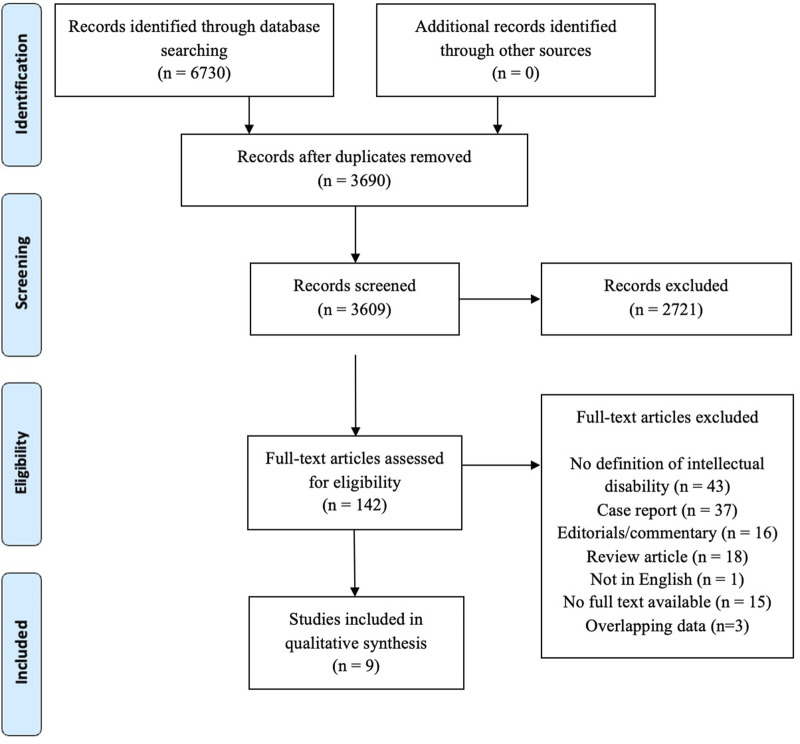
PRISMA flow chart.

3,690 records (Figure 1) were screened based on title and abstract, after removal of duplicates. A total of 142 full texts were assessed and finally nine studies were included for quantitative synthesis [16–24]. Three studies were excluded since a more recent study provided an update of previous data [24–27]. One study presented data on kidney, heart and liver transplantation [24] whereas the other studies presented data on either kidney (n = 5) [16–18, 21, 22] or heart (n = 3) [19, 20, 23] transplantation. All studies included patients with ID and patients without ID. Three studies were single-centered [16–18], whereas the other studies were multicentered. Three studies presenting data on heart transplantation and two studies on kidney transplantation likely used, in part, duplicate data from registries (UNOS/OPTN/Medicare) with overlapping inclusion periods between 2004 and 2017 [20, 21, 23, 24].

### Quality Assessment

**FIGURE 2:**
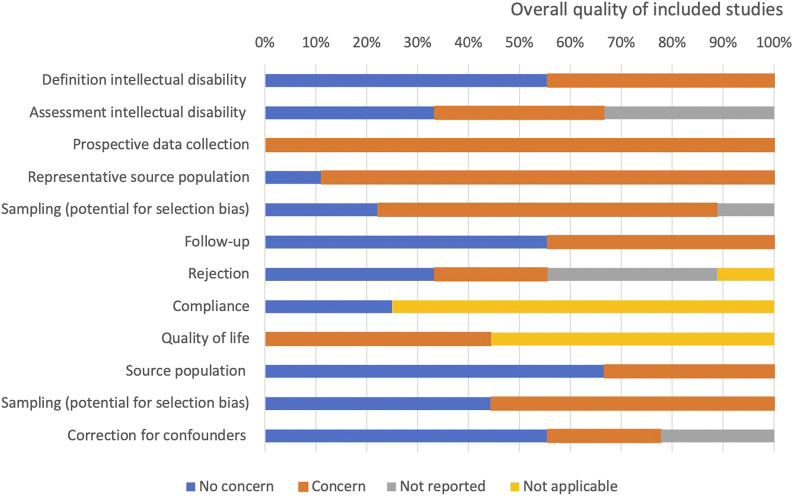
Quality assessment of the individual studies.

Quality assessment of individual studies and of the entire review sample is summarized in Table 1 and Figure 2. All studies were observational studies, collecting their data from patient charts or prospectively maintained registry databases. Definition of ID was clearly stated by five studies [16–18, 21, 22], and three of them commented appropriately on the assessment of ID [16, 17, 22]. Eight out of nine studies are at risk for selection bias as the studied populations may not represent the entire source population of patients with ID assessed or waitlisted for transplantation [16–20, 22–24]. Additionally, most studies were at risk of bias related to sampling of the population [16, 19, 20, 22–24]. Adequate follow-up periods (i.e., median follow-up above 36 months) were described by five studies [16, 17, 19, 23, 24]. Definitions of outcomes were infrequently provided. Five studies corrected results for potential confounding factors [16, 19, 21, 23, 24].

**TABLE 1:** Quality assessment of individual studies.

	Definition intellectual disability	Assessment of ID	Prospective data collection	Representative source population	Sampling (potential for selection bias)	Follow-up	Definition of rejection	Definition of compliance	Definition of quality of life	Controls from similar source population	Sampling controls (potential for selection bias)	Correction for confounders
Benedetti et al. [16]	1	1	3	3	3	1	3	1	3	1	3	1
Chen et al. [17]	1	1	3	3	1	1	2	4	4	1	1	2
Godown et al. [19]	3	2	3	3	3	1	1	4	3	3	3	1
Hand et al. [21]	1	2	3	1	1	3	1	4	4	1	1	1
Galante et al. [18]	1	2	3	3	2	3	2	1	4	3	3	3
Otha et al. [22]	1	1	3	3	3	3	1	1	3	3	3	3
Goel et al. [20]	3	3	3	3	3	3	2	4	4	1	3	2
Prendergast et al. [23]	3	3	3	3	3	1	4	4	4	1	1	1
Wightman et al. [24]	3	3	3	3	3	1	3	4	3	1	1	1

ID, intellectual disability, 1 no concerns, 2 not reported, 3 Any concern, 4 Not applicable.

### Study Characteristics

Supplementary Table A, B summarize the baseline characteristics of included studies. Studies were published between 1968 and 2023 in Japan (n = 1), Brazil (n = 1) or the USA (n = 7). Six out of nine studies (69%) included pediatric patients only [17, 19, 20, 22–24]. The other three studies included pediatric patients, young adults and adults with a maximum age of 49 [16, 18, 21]. Since outcome data for individual patients was not available, it was not possible to perform a sub-analysis on pediatric and adult patients. Various underlying disorders, such as genetic syndromes, congenital disorders, cerebral palsy, and developmental brain anomalies, were registered as cause of ID in all included studies. One study divided the study population in definite ID, probable ID and no-ID [20], whereas one study divided the patients into “within 1 grade level of peers,” “delayed grade level” or “in need of special education” [23].

### Definition and Assessment of ID

Two studies followed the definition of the American Association on Intellectual and Developmental Disabilities [17, 18] and two others the American Psychiatric Association definition [16, 22] (Table 2). Five studies used definitions that were not uniformly based on consensus guidelines or included registry data. Assessment of ID differed among the included studies. Two studies based their assessment on IQ, after assessment by a neuropsychologist [16, 22]. Another study assessed ID following the criteria of the DSM-5 or Bayley-II [17]. Three studies did not comment on the exact assessment of ID within the study population or used registry data [20, 23, 24].

**TABLE 2:** Definition of intellectual disability by included studies.

Study	Definition of ID	Assessment of ID
Benedetti et al. [16]	A significantly subaverage general intellectual functioning and concurrent deficits in adaptive functioning with onset prior to age 18. (American Psychiatric Association)	Standardized intelligence tests, IQ < 70, administered by a consultant neuropsychologist
Chen et al. [17]	Patient with severe deficits in multiple areas of function (adaptive, language, cognitive, motor, and self-care) who need a full-time caregiver irrespective of age based on definition of ID. (AAIDD)	Criteria from DSM-5 or Bayley II
Galante et al. [18]	Defined as stated by the AAIDD	ND
Goel et al. [20]	Definite ID: definite cognitive delay/impairment Probable ID: patients who met two of the three criteria: “probable” or “questionable” cognitive delay/impairment, “reduced academic load/non-participation,” or “delayed grade level/special education”	UNOS registry data was used, therefore assessment may vary
Godown et al. [19]	Patients with Down syndrome	ND
Hand et al. [21]	ICD codes for intellectual disability, pervasive developmental disorders, cerebral palsy or Down syndrome	ND
Prendergast et al. [23]	CD: DGL/need for special education/documented by provider as definite, probable, or questionable CD	OPTN registry data was used, therefore assessment may vary
Ohta et al. [22]	A significantly subaverage general intellectual functioning and concurrent deficits in adaptive functioning with onset prior to age 18 (American Psychiatric Association)	Intelligence quotient (IQ) or/and developmental quotient (DQ) by standardized intelligence tests as Wechsler. Intelligence Scale for Children-Third Edition or tests as Kyoto Scales of Psychological Development and Emoji Developmental Test
Wightman et al. [24]	Likert scales for (definite or probable) cognitive delay/impairment	UNOS registry data was used, therefore assessment may vary

ID, intellectual disability; AAIDD, American association on intellectual and developmental disabilities; CD, cognitive delay; IQ, intelligence quotient; DQ, development quotient; ICD, international classification of disease; OPTN, organ procurement and transplantation network; WGL, within 1 grade level of peers; DGL, delayed grade level; SE, special education; ND, Not described. ^#^Likert scales: 1, definite cognitive delay/impairment; 2, probable cognitive delay/impairment; 3, questionable cognitive delay/impairment; 4, no cognitive delay/impairment; and 5 not assessed.

### Selection of Patients With ID for Transplantation

The selection criteria of patients with ID for organ transplantation varied slightly between the studies. Four studies selected patients based on the reliability of their support network and the ability to take oral medication under supervision in order to minimize risk of rejection [16–18, 22]. Three studies did not specify how patients with ID were selected or excluded from organ transplantation [19, 23, 24]. One of the included studies evaluated a cohort of patients with end stage kidney disease. In this study, patients with ID were less likely to be evaluated for transplantation (OR: 0.46; 95% CI, 0.43–0.50) and less likely to be transplanted (OR: 0.38; 95% CI, 0.34–0.42) compared to propensity score matched patients without ID [21]. However, the latter study was based on registry data therefore criteria on which patients were selected remain unclear.

### Graft and Patient Survival

Reported graft and patient survival is summarized in Table 3, 4. Two studies on kidney transplantation and three studies on heart transplantation with potentially overlapping data, are shown in parallel [20, 21, 23, 24]. Reported graft survival was better or equal in patients with ID compared to control patients in seven out of nine studies. A study on heart transplantation reported a significantly lower graft survival in patients with delayed grade level compared to controls, whereas this was not reported for patients with special education [23]. Patient survival was reported to be equal in patients with ID compared to control patients in the majority of studies. Two studies reported significantly lower patient survival in kidney transplant recipients with ID compared to control patients. The study by Galante et al. reported significantly lower patient survival (survival at 5 years: ID 81%, n = 16 versus control: 97%, n = 83, *p* < 0.05). The larger registry based study by Wightman et al. reported significantly lower patient survival as well, although the actual reported survival difference was fairly minimal 95% versus 96% estimated survival at 10 years [18, 24].

**TABLE 3:** Graft survival after kidney, heart and liver transplantation in patients with intellectual disability and controls.

Graft survival	Sub group	N (ID)	N (Control)	1 year		3 years		5 years		10 years		P-value
				ID (%)	Control (%)	ID (%)	Control (%)	ID (%)	Control (%)	ID (%)	Control (%)	
Kidney transplantation												
Benedetti et al. [16]		8	100	100	86	ND		100	66	ND		0.04
Chen et al. [17]		10	62	100	88	100	80	100	77	ND		NS
Galante et al. [18]		16	83	88	94	81.2	88	81.2	80.2	73	70	NS
Ohta et al. [22]		25	164	100	95	ND		100	87	ND		NS
Hand et al. [21]		629	629	ND	ND^a^	ND		ND		ND		NS
Wightman et al. [24]^		594	5,643	98	97	ND		93	85	71	64	<0.01
Heart transplantation												
Goel et al. [20]	Def ID	131	1,959	88	91	84	84	ND		ND		NS
	Prob ID	434	1,959	91	91	82	84	ND		ND		NS
Prendergast et al. [23]	DGL	269	1,707	95	97	88	90	77	85	ND		<0.001
	SE	269	1,707	97	97	93	90	89	85	ND		NS
Wightman et al. [24]^		324	2,762	99	85	ND	ND	94	92	75	85	NS
Liver transplantation												
Wightman et al. [24]^		318	3679	93	95	ND	ND	92	92	92	87	NS

ID, intellectual disability; Def, definite; Prob, probable; DGL, delayed grade level; SE, special education; ND, not described; NS, no significant difference.

^a^ Exact numbers for ID and control not provided, overall >98%, ^ = death-censored graft survival.

**TABLE 4:** Patient survival after kidney, heart and liver transplantation in patients with ID and controls.

Patient survival	Sub group	N (ID)	N (Control)	1 year		3 years		5 years		10 years		P-value
				ID (%)	Control (%)	ID (%)	Control (%)	ID (%)	Control (%)	ID (%)	Control (%)	
Kidney transplantation												
Benedetti et al. [16]		8	100	100	97	ND		100	94	ND		NS
Chen et al. [17]		10	62	ND		100	98	ND		ND		NS
Galante et al. [18]		16	83	87	100	81	100	81	97	72	97	<0.05
Ohta et al. [22]		25	164	100	98	ND		100	98	ND		NS
Hand et al. [21]		629	629	ND	ND^a^	ND		ND		ND		NS
Wightman et al. [24]		594	5,643	99	99	ND		96	98	95	96	<0.01
Heart transplantation												
Goel et al. [20]	Def ID	131	1,959	89	92	86	86	ND		ND		NS
	Prob ID	434	1,959	92	92	82	86					NS
Godown et al. [19]		23	ND	100	ND	92	ND	92	ND	92	ND	NS
Wightman et al. [24]		324	2,762	95	92	ND	ND	86	83	73	72	NS
Liver transplantation												
Wightman et al. [24]		318	3,679	96	95	ND	ND	91	92	85	90	NS

ID, intellectual disability; Def, definite; Prob, probable; *, *p* < 0.05; n, population; NS, no significant difference; ND, not described.

^a^ Exact numbers for ID and control not provided, overall >98%.

### Treatment Adherence

Three studies (including 369 patients) presented data on medication adherence [16, 18, 22]. The criteria for non-adherence to the overall treatment process included cyclosporine or tacrolimus levels below 30 ng/mL or 1.5 ng/mL, >20% missed clinical visits and/or a post-transplantation weight gain of more than 20% above ideal body weight. All three studies reported complete treatment adherence (i.e. 100%, n = 49) amongst patients with ID. In two studies including a control group, adherence rates were 94% (n = 83) and 100% (n = 164) in patients without ID [18, 22].

### Acute Rejection

Data on acute rejection was reported by 8 out of 9 studies and is summarized in Table 5. Definitions of rejection were reported by six studies [16–19, 21, 22] and were defined as biopsy proven or the need to adjust the immunosuppression regimen. Two studies were based on registry data and therefore used the definition as provided by UNOS [20, 24]. None of the included studies reported a significant difference in incidences of acute rejection in patients with and without ID.

**TABLE 5:** Rejection in intellectual disability versus no intellectual disability per organ transplantation.

Study	Definition of rejection	Intellectual disability	Control	P-value
Kidney transplantation				
Chen et al. [17]	Biopsy proven rejection	1/10 (11%)	17/62 (27%)	0.29
Galante et al. [18]	Rejection-free survival	7/16 (75%)	24/83 (67%)	0.79
Ohta et al. [22]	Clinically manifested and treated rejection	7/25 (28%)	61/164 (37%)	0.40
Benedetti et al. [16]	Biopsy proven rejection	4/8 (50%)	46/100 (46%)	0.38
Hand et al. [21]	ICD-10 code T68.11 (since 2015) corresponding to graft rejection	50/629 (8.0%)	47/629 (7.5%)	NS
Wightman et al. [24]	UNOS definition	101/594 (17%)	1,524/5,643 (27%)	NS
Heart transplantation				
Goel et al. [20]	UNOS definition	Def ID: 22/131 (24%) Prob ID: 57/434 (18%)	295/1959 (20%)	Def ID: 0.207 Prob ID: 0.354
Godown et al. [19]	Clinical event, biopsy confirmed or not, that prompted augmentation of immunosuppression regimen	10/23 (43%)	ND	0.77
Wightman et al. [24]	UNOS definition	42/324 (13%)	249/2,762 (9%)	NS
Liver transplantation				
Wightman et al. [24]	UNOS definition	32/318 (10%)	405/3,679 (11%)	NS

Def, definitive; Prob, probable; n, population; p, *p*-value; NS, no significant difference; ND, not described.

### Quality of Life

Quality of life was assessed in four studies [16, 19, 22, 24]. Nearly all patients receiving a kidney transplant were on peritoneal dialysis or hemodialysis prior to transplantation. One study described an increase in quality of life in all patients and in 60% of the main caregivers [22]. Another study found that 100% of the main caregivers expressed the opinion that the patients’ quality of life had improved compared to dialysis [16]. Both studies used caregiver reported outcome measures rating the patient’s quality of life on a five-point Likert scale and comparing potential impact of kidney transplantation. None of these changes in quality of life have been compared to scores in controls. A study concerning heart transplantation scored the functional status post-transplantation of the patients according to the assistance needed in daily activities and found similar values pre- and post-transplantation. These results were not compared to a control group [19]. The study including patients with kidney, liver and heart transplantation presented data on functional status and found an improvement of 90%–100% post-transplantation in all groups [24].

## Discussion

This study provides a systematic overview of available literature on the outcomes after solid organ transplantation in patients with ID, compared to patients without ID. Graft and patient survival was not impaired in patients with ID in the majority of reports. Although varying definitions were used, acute rejection rates were not increased in patients with ID. Available studies do not suggest a substantial deficit in treatment adherence in patients with ID. Quality-of-life post-transplantation was studied in nearly half of the included studies. Although using various scoring tools, transplantation appears associated with improved quality of life in patients with ID. Among included studies both the definition and assessment of ID differed substantially or was not fully described. One study assessed patients with end-stage kidney disease and found the chances to be evaluated for transplantation and to actually receive a transplantation to be significantly lower (54% and 62% respectively) in patients with ID as compared to matched control patients [21]. Also, if pre-transplant selection criteria were reported, it was unclear what the criteria, such as ‘sufficient support network’ were. More data is required to detail the support network of the patients with ID, the amount of self-support, and their health status before transplantation.

Results of solid organ transplantation in patients with ID appear favorable, reporting adequate survival, adherence, and improved quality of life when an adequate support network is present. This is in accordance with a prior review from Wightman et al. [28], which included in addition disease-specific case studies on disorders variably causing ID. Another report from Thom et al. [29] supports this conclusion and discussed the ethical and legal aspects of the access to organs for patients with impaired decision making capacity. Current perceptions on ID being a relative or absolute contra-indication for organ transplantation are not ethically justifiable. Allocation of organs should be based on outcomes of transplantation in patients with and without ID rather than ethical considerations about benefit, utility, and fairness.

As quality of life is not routinely measured or considered in organ allocation, the relativity and subjectivity of such argumentation in the current context is emphasized. Societal and ethical values or impact are even more complex to quantify. For detailed ethical considerations we would refer to the excellent review written by Wightman et al., who concluded that exclusion based on intellectual disability would not be defensible from a societal and ethical perspective, and the recent recommendations by Thom et al. [6, 29]. In order to prevent discrimination of patients with ID and reach consensus among transplantation centers, it is important to define specific legislation. In North America, this is currently being developed, with the most recent being the introduction of the Charlotte Woodward Organ Transplant Discrimination Prevention Act to the senate of the United States [30], which prohibits to deny or restrict individual access to organ transplants solely on the basis of ID. In Europe, the European Disability Strategy was launched in 2021 by the European Union in order to protect the rights of people with disabilities [31]. The rising number of laws have also evoked criticism because interpretation in practice can still be highly ambiguous [32]. In a survey study from Richards et al. more than half of the included transplant programs report that informal processes guide the use of neurodevelopmental delay in the decision of listing a patient for transplantation and thereby emphasizes the lack of clinical implementation [33]. Some say rather than legislation, the field could benefit from unambiguous definition of the meaning and role of disability for consideration for solid organ transplantation [34]. An interesting approach is the social model of disability, proposed by Sara Goering, that describes how social norms can be disabling, rather than the objective impairment itself [35]. For example, the presumption that disability indicates a decreased quality of life may not be how intellectual disabled patients experience this themselves. Listening to these experiences can challenge how clinicians understand disability and its role considering scarce resources. Additionally, a more pragmatic perspective on this matter was studied by Freiberger et al. [36] at the Boston Children’s Hospital Center by assigning an advisory committee to ensure transplant selection criteria were nondiscriminatory. Data showed that amongst race and socioeconomic factors, patients with a severe neurodevelopmental delay had a significantly lower chance of being listed compared to controls. The suggested institutional committees can fill in the gaps between law and practice, and provide solutions were possible. Although more international data on decision making, listing and quality of life after transplantation is needed to ensure fair distribution of transplant organs, regional initiatives, as seen in Boston, show a valuable contribution to this matter.

### Limitations

This systematic literature review has several limitations. Included studies focused mainly on post-transplant outcomes, little data is provided on patients with ID on the transplant waitlist or patients with end stage organ failure not considered for transplantation. Therefore, it remains unclear how large the total population of patients with ID and end-stage organ failure is in need for organ transplantation. In addition, a selection bias of patients with an adequate support network and therefore suspected sufficient adherence may have occurred, resulting in favorable outcomes. Nevertheless, it may also be argued that adherence in patients lacking decisional capacity is mostly higher due to engagement of caregivers [29]. Three included studies on heart transplantation and two studies on kidney transplantation used registry databases with overlapping inclusion periods [20, 21, 23, 24]. Unfortunately, varying definitions of ID were used, and severity of ID was usually not considered. Generally, studies were small or presented a low level of detail, used unclear or wide definitions, and assessment methods were often unstandardized or subjective, therefore pooled analysis was not possible.

### Conclusion

Based on the current available literature, albeit of suboptimal methodological quality and limited scope, there is no evidence to support views that intellectual disability should in and of itself be considered a contra-indication for solid organ transplantation. Our results support the recommendations stating that specific international guidelines and their translation to clinical practice are necessary to prevent discrimination based on intellectual disability in the allocation of organs. Solid organ transplantation in patients with intellectual disability may have predominantly been performed in patients with a network available to support management and treatments required when living with a donor organ. In these patients, outcomes appear satisfactory and do not suggest lack of adherence or insufficient improvement in quality of life, although more data is needed to validate these conclusions.
