# Supplementary material for: Outcome of Solid Organ Transplantation in Patients With Intellectual Disability: A Systematic Literature Review
Source: Transpl Int. 2024 Oct 17;37:11872. doi: 10.3389/ti.2024.11872 (PMC11524806; doi:10.3389/ti.2024.11872)
Supplement: Supplementary file 1 [file DataSheet2.docx]

**Supplementary B: Table A, B and C: Baseline characteristics of included studies**

| **Part A of Baseline Characteristics** | | | | | | | | | | | | |
| --- | --- | --- | --- | --- | --- | --- | --- | --- | --- | --- | --- | --- |
| **Organ** | **Kidney** | | | | | | | | | | | |
| **Author and publication year** | Ohta et al. 2016 | | Benedetti et al. 1998 | | Chen et al. 2017 | | Galante et al. 2010 | | Hand et al. 2023 | | Wightman et al. 2021 | |
| **Study design** | Retrospective cohort study (1998-2004) | | Retrospective cohort study (1968-1996) | | Retrospective cohort study (2002-2012) | | Retrospective cohort study (1997-2006) | | Retrospective cohort study (2013-2020) | | Retrospective cohort study from UNOS registry (2008-2017) | |
| **Type of patient** | ID | No ID | ID | No ID | ID | No ID | ID | No ID | ID | No ID | ID | No ID |
| **N** | 25 | 164 | 8 | 100 | 10 | 62 | 16 | 83 | 692 | 692 | 594 | 5643 |
| **Age (years)** | 10.0 ± 5.3 | 11.4 ± 4.6 | 24 | - | <12: 70% >12: 30% | <12: 35% >12: 65% | 15±8.7 | 20±9.8 | 37 (29-49) | 38 (29-49) | 47% 11-17 | 59% 11-17 |
| **Male** | 36% | 60% | 88% | - | - | - | 75% | 52% | 33% | 33% | 59% | 58% |
| **Ethnicity** | - | - | 50% White;  25% Black; 25% Hispanic | - | 70% White;  10% Black; 20% Hispanic; | 56% White;  35% Black 8% Hispanic; | - | - | 65% White;  22% Black;  14% Other |  | 52%  White;  17%  Black;  24%  Hispanic;  8%  Other | 49%  White;  18%  Black;  27%  Hispanic;  6%  Other |
| **Average IQ** | 42 | - | 56 | - | - | - | - | - | - | - | - | - |
| **Intellectual disability diagnosis** | 40% Genetic syndromes  24% Other or unknown causes 20% Chromosome aberrations 8% Developmental brain anomaly 4% Perinatal causes  4% Acquired diseases | | 63% Congenital  13% Down Syndrome 13% Fragile X syndrome 13% Di George syndrome | | 30% Cerebrovascular accident 10% Joubert syndrome  10% Hypoxic ischemic encephalopathy 10% Foetal drugs and alcohol exposure 20% Prematurity 10% Down syndrome  10% Dyskeratosis congenita | | 25% Down syndrome  25% Developmental brain anomaly  19% Cerebral palsy 6% Duplication chromosome 2 6% Robinow syndrome 6% Lesch-Nyhan syndrome  6% Wilson's disease 6% Monosomy of chromosome 9 | | 10% Cerebral palsy  6% Down syndrome  Intellectual disability:  - 10% Mild  - 3% Moderate  - 4% Profound  - 31% other  Pervasive developmental disorders  - 3% Autism  - 2% Other | | - | |
| **Follow-up (median)** | 20 months | | 7.3 years | | 5.3 years | | 6.4 years | | 1 year | | 10 years | |

| **Part B of Baseline Characteristics** | | | | | | | | | | | | |
| --- | --- | --- | --- | --- | --- | --- | --- | --- | --- | --- | --- | --- |
| **Organ** | **Heart** | | | | | | | | | | **Liver** | |
| **Author and publication year** | Goel et al. 2017 | | | Prendergast et al. 2017 | | | Godown et al. 2022 | | Wightman et al. 2021 | | Wightman et al. 2021 | |
| **Study design** | Retrospective cohort study from UNOS registry (2008-2015) | | | Retrospective cohort study from OPTN registry (2004-2014) | | | Retrospective cohort study (1992-2020) | | Retrospective cohort study from UNOS registry (2008-2017) | | Retrospective cohort study from UNOS registry (2008-2017) | |
| **Type of patient** | Def ID | Prob ID | No ID | DGL | SE | No ID | ID | No ID | ID | No ID | ID | No ID |
| **N** | 131 | 434 | 1959 | 269 | 269 | 1707 | 26 | ND | 324 | 2762 | 318 | 3679 |
| **Age (years)** | <1y 11% 1-4y 27% 5-12y 30% 13-18y 32% | <1y 35% 1-4y 32% 5-12y 17% 13-18y 16% | <1y 33% 1-4y 19% 5-12y 24% 13-18y 25% | 12 (6-15) | 10 (4-14) | 10  (4-14) | 10.4 (3-13.8) | ND | <1y 33%  1-5y 31%  6-10y 10%  11-17y 26% | <1y 29%  1-5y 23%  6-10y 14%  11-17y 34% | <1y 28%  1-5y 44%  6-10y 13%  11-17y 15% | <1y 28%  1-5y 38%  6-10y 13%  11-17y 20% |
| **Male** | 55% | 55% | 54% | 53% | 61% | 54% | 44% | ND | 63% | 55% | 61% | 49% |
| **Ethnicity** | 62% White; 22% Hispanic; 15% Black;  1% Other | 48% White;  23% Hispanic; 22% Black;  7% Other | 55% White; 21% Black; 17% Hispanic;  8% Other | 49% White ; 25% Black; 19% Hispanic | 53% White ; 24% Black ; 20% Hispanic | 54% White; 24% Black;  15% Hispanic | 62% White | ND | 55% White;  19% Black;  19% Hispanic;  6% Other | 52% White;  21% Black;  20% Hispanic;  6% Other | 46% White;  14% Black;  29% Hispanic;  13% Other | 52% White;  15% Black;  23% Hispanic;  10% Other |
| **Average IQ** | - | | | - | | | - | | - | | - | |
| **Intellectual disability diagnosis** | - | | | - | | | Down syndrome | | - | | - | |
| **Follow-up**  **(median)** | 3 years | | | 9 years | | | 2.8 years | | 10 years | | 10 years | |

*ID = Intellectual disability, DGL = Delayed grade level, SE = Special education, ND = not described, Def = definite, Prob = probable, - = no data*
