# Supplementary material for: Outcome of Solid Organ Transplantation in Patients With Intellectual Disability: A Systematic Literature Review
Source: Transpl Int. 2024 Oct 17;37:11872. doi: 10.3389/ti.2024.11872 (PMC11524806; doi:10.3389/ti.2024.11872)
Supplement: Supplementary file 2 [file DataSheet1.docx]

**Supplementary A Search syntax**

**Embase**

('intellectual impairment'/de OR 'mental deficiency'/exp OR 'trisomy 21'/de OR 'cognitive defect'/de OR 'postoperative cognitive dysfunction'/de OR (((congenital*) NEAR/3 (hypocupremia*)) OR Adrenoleukodystroph* OR Adrenomyeloneuropath* OR Melanodermic*-Leukodystroph* OR CdLS2 OR X-ALD OR idiocy OR mongolism* OR trisomy-21 OR Trisomy-G OR ((Glycogen-Storag* OR Pseudoglycogenos*) NEAR/3 (IIb OR 2 OR II OR IIs OR 2s OR Cardiomyopath*)) OR ((translocate*) NEAR/3 (15-21-22)) OR leprechaunism* OR mannosidos* OR ((intellect* OR IQ OR mental* OR cogniti* OR neurocogniti* OR X-Linked-Copper OR copper-transport* OR PDH OR PDHC OR Pyruvate-Dehydrogenas* OR Pyruvate-Decarboxylas* OR prolidase*) NEAR/3 (syndrome* OR disease* OR disorder* OR complex OR deficienc* OR retard* OR disabilit* OR incapacit* OR deficit* OR handicap* OR dysfunct* OR alter* OR defect* OR impairment* OR declin* OR deteriorat*)) OR ((Cri-du-Chat* OR Chromosom*-5 OR 5p OR cat-cry* OR Bronze-Schilder* OR Schilder-Addison* OR Down* OR mongol* OR Alper* OR coffin OR COFS OR Danon OR Dubowitz OR Ellis-van-Creveld OR Hunter* OR Hurler* OR Kabuki-makeup* OR leigh* OR maple-syrup-urine* OR marinesco* OR maroteaux* OR sandhoff* OR sanflippo* OR WAGR OR Weismann-netter* OR Lysosom* OR acrocallosal* OR Aicardi* OR Cockayne* OR Costello* OR de-Lange OR Typus-Degenerativ*-Amstelodamens* OR Siemerling-Creutzfeldt* OR Marker-X OR Martin-Bell* OR FRAXE OR FRAXA OR antopol* OR Danon OR Lesch-Nyhan OR fetal-alcohol* OR fetal-hydantoin* OR floating-harbor* OR fragile-X OR happy-puppet* OR infantile-Refsum* OR Lesch-Nyhan* OR Lowe* OR Mauriac* OR Menkes OR Menkea OR Steely-Hair OR kinky-hair OR Rett OR Prader-Willi* OR Sjoegren-Larsson* OR Smith-Lemli-Opitz* OR Smith-Magenis* OR Sotos* OR Williams OR Wolf-Hirschhorn* OR Zellweger*) NEAR/3 (syndrome* OR disease* OR disorder* OR complex))):ab,ti) AND ('heart transplantation'/exp OR 'kidney transplantation'/exp OR 'liver transplantation'/exp OR 'lung transplantation'/exp OR 'pancreas transplantation'/exp OR 'intestine transplantation'/exp OR (((transplant* OR graft* OR allograft* OR allotransplant* OR autograft* OR autotransplant* OR donor* OR engraft* OR isograft* OR isotransplant* OR retransplant* OR xenograft* OR xenotransplant*) NEAR/6 (heart* OR cardiac* OR kidney* OR kidnies* OR liver* OR renal* OR lung* OR pulmonar* OR pancreas* OR hepatic* OR intestin* OR small-bowel))):ab,ti) *NOT ([animals]/lim NOT [humans]/lim) NOT ([Conference Abstract]/lim) AND [english]/lim*

**Medline**

(Cognitive Dysfunction/ OR exp Intellectual Disability/ OR trisomy 21/ OR cognitive defect/ OR postoperative cognitive dysfunction/ OR (((congenital*) ADJ3 (hypocupremia*)) OR Adrenoleukodystroph* OR Adrenomyeloneuropath* OR Melanodermic*-Leukodystroph* OR CdLS2 OR X-ALD OR idiocy OR mongolism* OR trisomy-21 OR Trisomy-G OR ((Glycogen-Storag* OR Pseudoglycogenos*) ADJ3 (IIb OR 2 OR II OR IIs OR 2s OR Cardiomyopath*)) OR ((translocate*) ADJ3 (15-21-22)) OR leprechaunism* OR mannosidos* OR ((intellect* OR IQ OR mental* OR cogniti* OR neurocogniti* OR X-Linked-Copper OR copper-transport* OR PDH OR PDHC OR Pyruvate-Dehydrogenas* OR Pyruvate-Decarboxylas* OR prolidase*) ADJ3 (syndrome* OR disease* OR disorder* OR complex OR deficienc* OR retard* OR disabilit* OR incapacit* OR deficit* OR handicap* OR dysfunct* OR alter* OR defect* OR impairment* OR declin* OR deteriorat*)) OR ((Cri-du-Chat* OR Chromosom*-5 OR 5p OR cat-cry* OR Bronze-Schilder* OR Schilder-Addison* OR Down* OR mongol* OR Alper* OR coffin OR COFS OR Danon OR Dubowitz OR Ellis-van-Creveld OR Hunter* OR Hurler* OR Kabuki-makeup* OR leigh* OR maple-syrup-urine* OR marinesco* OR maroteaux* OR sandhoff* OR sanflippo* OR WAGR OR Weismann-netter* OR Lysosom* OR acrocallosal* OR Aicardi* OR Cockayne* OR Costello* OR de-Lange OR Typus-Degenerativ*-Amstelodamens* OR Siemerling-Creutzfeldt* OR Marker-X OR Martin-Bell* OR FRAXE OR FRAXA OR antopol* OR Danon OR Lesch-Nyhan OR fetal-alcohol* OR fetal-hydantoin* OR floating-harbor* OR fragile-X OR happy-puppet* OR infantile-Refsum* OR Lesch-Nyhan* OR Lowe* OR Mauriac* OR Menkes OR Menkea OR Steely-Hair OR kinky-hair OR Rett OR Prader-Willi* OR Sjoegren-Larsson* OR Smith-Lemli-Opitz* OR Smith-Magenis* OR Sotos* OR Williams OR Wolf-Hirschhorn* OR Zellweger*) ADJ3 (syndrome* OR disease* OR disorder* OR complex))).ab,ti.) AND (heart transplantation/ OR kidney transplantation/ OR liver transplantation/ OR lung transplantation/ OR pancreas transplantation/ OR intestine transplantation/ OR (((transplant* OR graft* OR allograft* OR allotransplant* OR autograft* OR autotransplant* OR donor* OR engraft* OR isograft* OR isotransplant* OR retransplant* OR xenograft* OR xenotransplant*) ADJ6 (heart* OR cardiac* OR kidney* OR kidnies* OR liver* OR renal* OR lung* OR pulmonar* OR pancreas* OR hepatic* OR intestin* OR small-bowel))).ab,ti.) *NOT (exp animals/ NOT humans/) NOT (news OR congres* OR abstract* OR book* OR chapter* OR dissertation abstract*).pt.* *AND english.la.*

**PsycINFO**

(Cognitive Dysfunction/ OR exp Intellectual Disability/ OR trisomy 21/ OR cognitive defect/ OR postoperative cognitive dysfunction/ OR (((congenital*) ADJ3 (hypocupremia*)) OR Adrenoleukodystroph* OR Adrenomyeloneuropath* OR Melanodermic*-Leukodystroph* OR CdLS2 OR X-ALD OR idiocy OR mongolism* OR trisomy-21 OR Trisomy-G OR ((Glycogen-Storag* OR Pseudoglycogenos*) ADJ3 (IIb OR 2 OR II OR IIs OR 2s OR Cardiomyopath*)) OR ((translocate*) ADJ3 (15-21-22)) OR leprechaunism* OR mannosidos* OR ((intellect* OR IQ OR mental* OR cogniti* OR neurocogniti* OR X-Linked-Copper OR copper-transport* OR PDH OR PDHC OR Pyruvate-Dehydrogenas* OR Pyruvate-Decarboxylas* OR prolidase*) ADJ3 (syndrome* OR disease* OR disorder* OR complex OR deficienc* OR retard* OR disabilit* OR incapacit* OR deficit* OR handicap* OR dysfunct* OR alter* OR defect* OR impairment* OR declin* OR deteriorat*)) OR ((Cri-du-Chat* OR Chromosom*-5 OR 5p OR cat-cry* OR Bronze-Schilder* OR Schilder-Addison* OR Down* OR mongol* OR Alper* OR coffin OR COFS OR Danon OR Dubowitz OR Ellis-van-Creveld OR Hunter* OR Hurler* OR Kabuki-makeup* OR leigh* OR maple-syrup-urine* OR marinesco* OR maroteaux* OR sandhoff* OR sanflippo* OR WAGR OR Weismann-netter* OR Lysosom* OR acrocallosal* OR Aicardi* OR Cockayne* OR Costello* OR de-Lange OR Typus-Degenerativ*-Amstelodamens* OR Siemerling-Creutzfeldt* OR Marker-X OR Martin-Bell* OR FRAXE OR FRAXA OR antopol* OR Danon OR Lesch-Nyhan OR fetal-alcohol* OR fetal-hydantoin* OR floating-harbor* OR fragile-X OR happy-puppet* OR infantile-Refsum* OR Lesch-Nyhan* OR Lowe* OR Mauriac* OR Menkes OR Menkea OR Steely-Hair OR kinky-hair OR Rett OR Prader-Willi* OR Sjoegren-Larsson* OR Smith-Lemli-Opitz* OR Smith-Magenis* OR Sotos* OR Williams OR Wolf-Hirschhorn* OR Zellweger*) ADJ3 (syndrome* OR disease* OR disorder* OR complex))).ab,ti.) AND (heart transplantation/ OR kidney transplantation/ OR liver transplantation/ OR lung transplantation/ OR pancreas transplantation/ OR intestine transplantation/ OR (((transplant* OR graft* OR allograft* OR allotransplant* OR autograft* OR autotransplant* OR donor* OR engraft* OR isograft* OR isotransplant* OR retransplant* OR xenograft* OR xenotransplant*) ADJ6 (heart* OR cardiac* OR kidney* OR kidnies* OR liver* OR renal* OR lung* OR pulmonar* OR pancreas* OR hepatic* OR intestin* OR small-bowel))).ab,ti.) *NOT (exp animals/ NOT humans/) NOT (news OR congres* OR abstract* OR book* OR chapter* OR dissertation abstract*).pt.* *AND english.la.*

**Cochrane (RCTs)**

((((congenital*) NEAR/3 (hypocupremia*)) OR Adrenoleukodystroph* OR Adrenomyeloneuropath* OR (Melanodermic* NEXT/1 Leukodystroph*) OR CdLS2 OR (X NEXT/1 ALD) OR idiocy OR mongolism* OR (trisomy NEXT/1 21) OR (Trisomy NEXT/1 G) OR ((Glycogen NEXT Storag* OR Pseudoglycogenos*) NEAR/3 (IIb OR 2 OR II OR IIs OR 2s OR Cardiomyopath*)) OR leprechaunism* OR mannosidos* OR ((intellect* OR IQ OR mental* OR cogniti* OR neurocogniti* OR X NEXT Linked NEXT Copper OR copper NEXT transport* OR PDH OR PDHC OR Pyruvate NEXT Dehydrogenas* OR Pyruvate NEXT Decarboxylas* OR prolidase*) NEAR/3 (syndrome* OR disease* OR disorder* OR complex OR deficienc* OR retard* OR disabilit* OR incapacit* OR deficit* OR handicap* OR dysfunct* OR alter* OR defect* OR impairment* OR declin* OR deteriorat*)) OR (((Cri NEXT du NEXT/1 Chat*) OR (Chromosom* NEXT/1 5) OR 5p OR (cat NEXT/1 cry*) OR (Bronze NEXT/1 Schilder*) OR (Schilder NEXT/1 Addison*) OR Down* OR mongol* OR Alper* OR coffin OR COFS OR Danon OR Dubowitz OR Ellis NEXT van NEXT Creveld OR Hunter* OR Hurler* OR (Kabuki NEXT/1 makeup*) OR leigh* OR (maple NEXT syrup NEXT/1 urine*) OR marinesco* OR maroteaux* OR sandhoff* OR sanflippo* OR WAGR OR (Weismann NEXT/1 netter*) OR Lysosom* OR acrocallosal* OR Aicardi* OR Cockayne* OR Costello* OR de NEXT Lange OR (Typus NEXT Degenerative NEXT/1 Amstelodamens*) OR (Siemerling NEXT/1 Creutzfeldt*) OR Marker NEXT X OR (Martin NEXT/1 Bell*) OR FRAXE OR FRAXA OR antopol* OR Danon OR Lesch NEXT Nyhan OR (fetal NEXT/1 alcohol*) OR (fetal NEXT/1 hydantoin*) OR (floating NEXT/1 harbor*) OR fragile NEXT X OR (happy NEXT/1 puppet*) OR (infantile NEXT/1 Refsum*) OR (Lesch NEXT/1 Nyhan*) OR Lowe* OR Mauriac* OR Menkes OR Menkea OR Steely NEXT Hair OR kinky NEXT hair OR Rett OR (Prader NEXT/1 Willi*) OR (Sjoegren NEXT/1 Larsson*) OR (Smith NEXT Lemli NEXT/1 Opitz*) OR (Smith NEXT/1 Magenis*) OR Sotos* OR Williams OR (Wolf NEXT/1 Hirschhorn*) OR Zellweger*) NEAR/3 (syndrome* OR disease* OR disorder* OR complex))):ab,ti) AND ((((transplant* OR graft* OR allograft* OR allotransplant* OR autograft* OR autotransplant* OR donor* OR engraft* OR isograft* OR isotransplant* OR retransplant* OR xenograft* OR xenotransplant*) NEAR/6 (heart* OR cardiac* OR kidney* OR kidnies* OR liver* OR renal* OR lung* OR pulmonar* OR pancreas* OR hepatic* OR intestin* OR small NEXT bowel))):ab,ti)

**Web of Science**

TS=(((((congenital*) NEAR/2 (hypocupremia*)) OR Adrenoleukodystroph* OR Adrenomyeloneuropath* OR Melanodermic*-Leukodystroph* OR CdLS2 OR (X NEAR/1 ALD) OR idiocy OR mongolism* OR trisomy-21 OR Trisomy-G OR ((Glycogen-Storag* OR Pseudoglycogenos*) NEAR/2 (IIb OR 2 OR II OR IIs OR 2s OR Cardiomyopath*)) OR leprechaunism* OR mannosidos* OR ((intellect* OR IQ OR mental* OR cogniti* OR neurocogniti* OR X-Linked-Copper OR copper-transport* OR PDH OR PDHC OR Pyruvate-Dehydrogenas* OR Pyruvate-Decarboxylas* OR prolidase*) NEAR/2 (syndrome* OR disease* OR disorder* OR complex OR deficienc* OR retard* OR disabilit* OR incapacit* OR deficit* OR handicap* OR dysfunct* OR alter* OR defect* OR impairment* OR declin* OR deteriorat*)) OR ((Cri-du-Chat* OR Chromosom*-5 OR 5p OR cat-cry* OR Bronze-Schilder* OR Schilder-Addison* OR Down* OR mongol* OR Alper* OR coffin OR COFS OR Danon OR Dubowitz OR Ellis-van-Creveld OR Hunter* OR Hurler* OR Kabuki-makeup* OR leigh* OR maple-syrup-urine* OR marinesco* OR maroteaux* OR sandhoff* OR sanflippo* OR WAGR OR Weismann-netter* OR Lysosom* OR acrocallosal* OR Aicardi* OR Cockayne* OR Costello* OR de-Lange OR Typus-Degenerative-Amstelodamens* OR Siemerling-Creutzfeldt* OR Marker-X OR Martin-Bell* OR FRAXE OR FRAXA OR antopol* OR Danon OR Lesch-Nyhan OR fetal-alcohol* OR fetal-hydantoin* OR floating-harbor* OR fragile-X OR happy-puppet* OR infantile-Refsum* OR Lesch-Nyhan* OR Lowe* OR Mauriac* OR Menkes OR Menkea OR Steely-Hair OR kinky-hair OR Rett OR Prader-Willi* OR Sjoegren-Larsson* OR Smith-Lemli-Opitz* OR Smith-Magenis* OR Sotos* OR Williams OR Wolf-Hirschhorn* OR Zellweger*) NEAR/2 (syndrome* OR disease* OR disorder* OR complex)))) AND ((((transplant* OR graft* OR allograft* OR allotransplant* OR autograft* OR autotransplant* OR donor* OR engraft* OR isograft* OR isotransplant* OR retransplant* OR xenograft* OR xenotransplant*) NEAR/5 (heart* OR cardiac* OR kidney* OR kidnies* OR liver* OR renal* OR lung* OR pulmonar* OR pancreas* OR hepatic* OR intestin* OR small-bowel)))) *NOT ((animal* OR rat OR rats OR mouse OR mice OR murine OR dog OR dogs OR canine OR cat OR cats OR feline OR rabbit OR cow OR cows OR bovine OR rodent* OR sheep OR ovine OR pig OR swine OR porcine OR veterinar* OR chick* OR zebrafish* OR baboon* OR nonhuman* OR primate* OR cattle* OR goose OR geese OR duck OR macaque* OR avian* OR bird* OR fish*) NOT (human* OR patient* OR women OR woman OR men OR man))*) *AND DT=(Article OR Review) AND LA=(English)*

**Google Scholar – most relevant top-200**

mongolism|"intellect|mental|cognitive|cognition syndrome|disease|disorder|deficiency|retardation|impairment" "transplant|transplantation heart|cardiac|kidney|kidnies|liver|renal|lung|pulmonary|pancreas|hepatic|intestine"
